# Clomiphene citrate treatment for late onset hypogonadism: rise and fall

**DOI:** 10.1590/S1677-5538.IBJU.2016.0112

**Published:** 2016

**Authors:** Marcelo Marconi, Renato Souper, Jonathan Hartmann, Matías Alvarez, Ignacio Fuentes, Francisco J. Guarda

**Affiliations:** 1 Departamento de Urología de la Universidad Católica de Chile, Santiago, Chile; 2 Facultad de Medicina, Universidad Católica de Chile, Santiago, Chile; 3 Departamento de Endocrinología, Universidad Católica de Chile, Santiago, Chile

**Keywords:** Hypogonadism, Clomiphene, Testosterone, Therapeutics

## Abstract

**Objective:**

Previous series have demonstrated that Clomiphene Citrate (CC) is an effective treatment to increase Total Testosterone (TT) in Late Onset Hypogonadism (LOH) patients. However, what happens to TT levels after ending CC treatment is still debatable. The objective of this study is to evaluate TT levels 3 months after the discontinuation of CC in patients with LOH who were previously successfully treated with the same drug.

**Materials and Methods:**

Twenty-seven patients with LOH that were successfully treated (achieved TT levels >11nmol/l) with CC 50mgs daily for 50 days were prospectively recruited in our Andrological outpatient clinic. CC was then stopped for 3 months and TT levels were measured at the end of this period.

**Results:**

Mean TT level before discontinuation of CC was 22.7±8.1nmol/L (mean±SD). Three months after discontinuation, mean TT level significantly decreased in all patients, 10.2±3.9nmol/l (p<0.01). Twenty-one patients (78%) decreased TT levels under 11nmol/L. Six patients (22%) had TT levels that remained within the normal recommended range (≥11nmol/l). No statistical significant differences were observed between both groups.

**Conclusion:**

In the short term LOH does not seem to be a reversible condition in most patients after CC treatment. More studies with longer follow-up are needed to evaluate the kinetics of TT in LOH.

## INTRODUCTION

The exact prevalence of Late Onset Hypogonadism (LOH) is a matter of debate (1–3); however, there is consensus that it constitutes an emerging problem (4). Testosterone Replacement Therapy (TRT) using different formulations i.e. intramuscular, transcutaneous and trans-mucosal-among others-is the most popular treatment strategy for LOH (5). Based in clinical experience and following recommendations coming from guidelines, in most patients the indication of TRT may be clear; however, in some cases it may be controversial or even contraindicated. Examples of these situations are patients who want to father a child in the near future, in which TRT is contraindicated (6), patients with LOH symptoms-i.e. decreased sexual interest, erectile dysfunction-and Total Testosterone (TT) levels that are under but very close to the normal recommended range (≥11nmoL/L), in whom the clinician may doubt whether the symptoms are really related to TT levels (7) or may be related to other conditions-i.e. psychological issues; and finally, men that may have transient hypogonadism (8, 9), for example due to stressful conditions (10). In these cases, but also in typical LOH patients, Clomiphene Citrate (CC) has become an extremely interesting alternative (11). Clomiphene Citrate blocks the estrogen receptor in the hypothalamus and pituitary gland, increasing FSH and LH levels and secondarily increasing spermatogenesis and testosterone levels (11, 12). Even though, Clomiphene Citrate treatment is not approved for men in many countries, it has been used over-the-counter for decades, first to improve sperm count and in the last 15 years it has proved to be an effective and safe strategy to increase testosterone levels in patients with LOH (13, 14). Clomiphene Citrate has advantage of not affecting fertility and not blocking the Hypothalamus-Pituitary-Testis (HPT) axis. However, information about CC treatment for LOH is still scarce, especially regarding the kinetics of TT levels and the potential recovery of HPT axis after treatment discontinuation. Taking this information into account, the objective of this study is to evaluate TT levels 3 months after the discontinuation of CC in patients with LOH who were previously successfully treated with the same drug.

## PATIENTS AND METHODS

Thirty patients (mean 50.1 years, range 32–70) with LOH were prospectively recruited in our Andrological outpatient clinic. Late Onset Hypogonadism was defined according to previous consensus definition (15):

- Symptoms: decreased sexual interest, decreased morning erections, erectile dysfunction.
- Total Testosterone <11nmol/l (in at least two different measurements) Regarding symptoms, 13/27 patients complained of decreased sexual desire, 11/27 with Erectile Dysfunction (ED), and 3/27 both symptoms. The median IIEF-5 score of all patients before treatment was 18 (range 11–24). In the subgroup that complained of exclusively ED or decreased sexual interest plus ED the median score was 15 (range 11–19).

To be eligible to be treated with CC, patients had normal FSH and LH, no thyroid function abnormalities neither hyperprolactinemia. After a 50-day treatment with 50mgs of CC daily, twenty-seven patients achieved normal TT levels (>11nmol/l). In this specific sub-group (n=27) CC was stopped for 3 months and TT levels were evaluated at the end of this period. Total testosterone was analyzed using electrochemiluminescence inmuno assay (Roche™). LH and FSH were measured using direct chemiluminimetric inmuno assay (Siemens™). The study was approved by the Research Ethical Committee of Pontificia Universidad Católica de Chile.

### Statistical analysis

Data analysis was performed using GraphPad™ software. Differences in TT levels before and after discontinuation of CC were analyzed with paired t-test, considering statistical significance with a p<0.05. Results are shown as mean±SD.

## RESULTS

**Figure 1:**
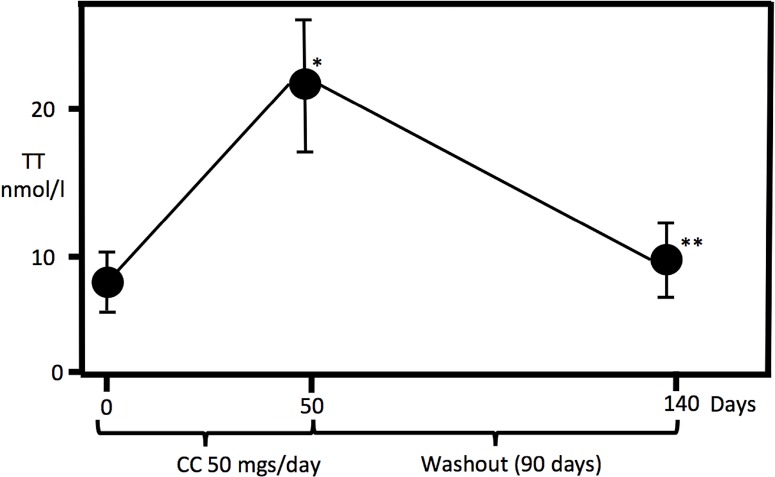
Total Testosterone (TT) levels at Day 0 (diagnosis), Day 50 (after Clomiphene citrate (cc) treatment, 50mgs. daily), and Day 140 (90 days after ending cc treatment). *p<0.01 between day 0 and day 50. **p<0.01 between day 50 and day 140.

Mean TT level at the time of diagnosis (n=30) was 8.5±1.8nmol/L (mean±SD), with normal range gonadotropins: FSH 5.1±4.9mIU/mL and LH 4.3±2.9mIU/mL. After a 50-day CC treatment, 27 patients achieved normal TT level. Mean TT level in this group was significantly higher than pre-treatment state, 22.7±8.1nmol/L, p<0.01 (Figure-1). Three months after discontinuation of treatment, mean TT level (n=27) was 10.2±3.9nmol/L. All patients (n=27) significantly decreased (p<0.01) TT after discontinuation of CC (Figure-1). Twenty-one (78%) patients decreased TT levels under 11nmol/L. Six patients (22%) had TT levels that remained within the normal recommended range (>11nmol/L) three months after discontinuation of CC. This subgroup (n=6) was controlled six months after discontinuation of CC, in all cases TT decreased to the pre-treatment levels (<11nmol/L). No statistical significant differences were observed between the group of patients who maintained TT levels within the normal range (three months after discontinuation) and those who decreased it (i.e. age, previous TT levels). Regarding symptoms, after increasing TT levels 38.5% (5/13) of patients who complained of decreased sexual desire improved the symptom, 27.3% (3/11) improved ED rising their IIEF-5 score (n=3) from a median of 18 (range 17–21) to 22 (21–24), and 2/3 (66.7%) improved both.

## DISCUSSION

Testosterone replacement therapy has significantly increased in the last decade (16), however, everyday practice concerns are multiple (17). First: TRT is meant to be a lifelong treatment, so the decision of starting therapy is considerable, especially regarding long term safety issues-i.e. cardiovascular, oncological, etc., costs for the patient and finally the fact that if the treatment is stopped, testosterone levels will drop and may become even lower than the one that motivated TRT due to HPT axis blockade. Second: the two cardinal symptoms that usually motivate TRT are decreased sexual desire and ED. We know that the first is very sensitive to TT levels (18), however, extremely subjective. On the other hand, ED is associated with TT levels much lower than the lower range in which TRT is indicated (7, 18), so it may be incorrectly associated with TT levels that are low, but not low enough to explain the symptom. Third: TRT produces reversible infertility, which constitutes a problem in patients who want to father a child. Fourth: we do not know how many patients may present only a transient hypogonadism which could recover TT levels after a “stimulation” treatment, meaning that an undetermined number of LOH cases may not need a chronic treatment but a short-term therapy or an intermittent one (8, 9).

Taking all this information into account, therapy with CC makes sense and evidence supports it. The results of our series demonstrate that 90% of men with LOH increased TT under treatment which agrees with other reports showing that in selected patients, CC is efficacious in a high proportion of cases and has the advantages of not blocking the HPT axis, not affecting fertility, not producing polycythemia, and all with lower costs than TRT (8, 19–21). Even an isomer of CC, Enclomiphene (EC), has been introduced recently, having the advantages of shorter half-life and theoretically more specificity to increase LH and FSH (22). EC has also demonstrated to increase testosterone levels in a high proportion of cases (23). So if CC or EC are such a good treatment, why not use it in all cases? And, if patients recover TT levels after a treatment with CC, is that recovery permanent once the therapy is cancelled?

Regarding the first question, it seems that one of the concerns would be the safety of the drug in the long term. Evidence suggests that long term CC treatment has no adverse effects and that efficacy is maintained, however, in the longer studies the follow-up does not exceed 46 months (14). In our study no adverse effects were reported; however, follow-up is too short to be conclusive.

The main objective of our study was to elucidate if LOH could be reversed by a 50-day CC treatment. Previous evidences were scarce, since most of the studies did not report the kinetics of TT after stopping CC treatment. We found three studies that reported TT level after ending treatment. Lim and cols. in 1976 reported in five hypogonadal uremic men an increase in TT levels after CC treatment that lasted for 12 months (24). Normal TT levels were reported 4 months after ending therapy. Guay and cols. in 2003 mentioned that in some patients with LOH, CC can be stopped and normal TT levels can be maintained (8). However, definitive data regarding this asseveration is lacking in the manuscript (8). Devoto and Aravena, reported that in a subgroup of patients with functional hypogonadotropic hypogonadism who respond to CC therapy, normal TT levels were maintained six months after ending treatment (9). On the other hand, Kaminetsky and cols, and Wiehle and cols. reported that in patients with secondary hypogonadism who responded to EC therapy, once the treatment was stopped TT levels decreased and returned to the pre-treatment values (23, 25). Our results concur with the last two authors since all patients decreased TT levels 3 months after discontinuation of CC treatment, 78% of them under the normal range. The six cases in which TT was in the normal range three months after CC discontinuation also dropped TT levels (to pre-treatment levels) six months after ending treatment. We think that our results reveal that LOH is a chronic irreversible condition in most cases. Since all cases dropped TT levels to pre-treatment levels we were not able to evaluate if certain patient characteristics (age, comorbidities, etc.) could predict a permanent response to CC treatment. The same occurred in the primary group (n=30) where 27 patients increased TT levels, making a comparison between responders and non-responders not statistically possible due to the small number of non-responder cases (n=3).

Considering our results and previous reports we think CC treatment would have three roles: First, it is an excellent alternative when patients are concerned about fertility, second, CC is an extraordinary alternative for a therapeutic individual trial in cases where we are not convinced that low testosterone is really the explanation to all its symptoms, especially when the TT level is very close to the normal ranges. Finally, CC represents a good alternative to TRT with the advantage of not blocking HPT axis, but taking into account our results and previous series (23, 25), it should be discussed with the patient that treatment will be permanent in most cases.

The main limitations of our study are: the short follow-up and the absence of the evaluation of estradiol and free testosterone. Regarding this last point, all patients had TT values under the normal range associated with symptoms, according to the guidelines in these cases TT would be enough for evaluation (5). Also no specific questionnaire was applied to the patients regarding LOH symptoms, however, these questionnaires are not recommended by the guidelines since their specificity is low and are not effective for case finding (5).

## CONCLUSIONS

Late Onset Hypogonadism on men who successfully respond to CC therapy in the short term do not seem to reverse the condition after ending treatment. More studies with longer follow-up are needed to evaluate the kinetics of TT in these patients.
